# A review of the genome, epidemiology, clinical features, prevention, and treatment scenario of COVID-19: Bangladesh aspects

**DOI:** 10.1186/s43168-021-00053-2

**Published:** 2021-01-29

**Authors:** Abdullah Al Noman, Md. Shofiqul Islam, Samiron Sana, Prapti Mondal, Rima Islam Meem, Sohel Rana, Debashish Mondol, Manoshi Sana, Sheikh I. Hossain, Taufique Joarder, Kishor Mazumder

**Affiliations:** 1 Department of Genetic Engineering & Biotechnology, Faculty of Biological Science and Technology, Jashore University of Science and Technology, Jashore, 7408 Bangladesh; 2 Pharmacy Discipline, Life Science School, Khulna University, Khulna, 9208 Bangladesh; 3 Department of Pharmacy, Faculty of Biological Science and Technology, Jashore University of Science and Technology, Jashore, 7408 Bangladesh; 4 Department of Zoology, Rajshahi College, Rajshahi, 6100 Bangladesh; 5 Dhaka Medical College, Dhaka, 1000 Bangladesh; 6 School of Mechanical and Mechatronic Engineering, University of Technology Sydney, 81 Broadway, Ultimo, NSW 2007 Australia; 7 Public Health Foundation, Dhaka, Bangladesh; 8 School of Biomedical Sciences, Charles Sturt University, Wagga, NSW 2678 Australia

**Keywords:** Bangladesh aspects, COVID-19, Genome, Epidemiology, Clinical features, Diagnosis, Treatment, Economic impact

## Abstract

**Background:**

The ongoing acute respiratory disease pandemic termed COVID-19 caused by a newly emerged coronavirus has jeopardized the world’s health and economic sectors. As of 20 July 2020, the virus now known as SARS-CoV-2 has already infected more than 14 million individuals and killed 612,815 patients with a mortality rate of 4.12% around the world regardless of age, gender, and race.

**Main body:**

Bangladesh has become one of its worst sufferers, with 207,453 infected people and 2668 related deaths with a mortality rate of 1.29% until 20 July 2020. More than 50% of COVID-19 patients in Bangladesh are aged between 21 and 40, but elderly people aged more than 60 have the highest mortality rate (44.7%). Male individuals are also more susceptible to the virus than females and consist of 71% and 79% among the infected and deceased patients, respectively. The most prevalent clinical features following the virus incubation period are fever, fatigue, and dry cough. A phylogenetic analysis study elucidated that the virus strain found in the country has 9 single-nucleotide variants, mostly in the ORF1ab gene, and a sequence containing 3 successive variants in the N protein, which reflects a weaker strain of SARS-CoV-2, implicating a possibility of its lower mortality rate. Another investigation of 184 genome samples of SARS-CoV-2 across the country implicated a close homology with a European haplotype of SARS-CoV-2. The country has also joined the race of vaccine development and started phase III clinical trials of a candidate vaccine developed by Sinovac Research and Development Co Ltd, China.

**Conclusion:**

Bangladesh, as a developing country, still struggles with the pandemic and needs to scale up its response operation and improve healthcare facilities such as testing capacity, institutional quarantine, and isolation centers and promote awareness. Preventive measures such as strict lockdown, social distancing, and boosting the existing immune system are thus considered the only contrivances.

## Background

In late December 2019, the world came to know about an unknown threat caused by a pathogen with unidentified etiology originating from a seafood market in Wuhan in Hubei Province, China, and the Chinese Center for Disease Control and Prevention (CCDC) proclaimed it as novel coronavirus pneumonia (NCP) [1]. The virus now known as severe acute respiratory syndrome coronavirus-2 (SARS-CoV-2) is included in the orthocoronavirinae subfamily, which belongs to the Coronaviridae family and is genetically grouped into main genera: *Alphacoronavirus* (α-CoV), *Betacoronavirus* (β-CoV), *Deltacoronavirus* (δ-CoV), and *Gammacoronavirus* (γ-CoV) [2]. Seven species of coronavirus, including the latest being SARS-CoV-2, have been identified as pathogenic for humans [3]. To date, two severe disease outbreaks associated with severe acute respiratory syndrome coronavirus (SARS-CoV) and Middle East respiratory syndrome coronavirus (MERS-CoV) have occurred by coronaviruses in China (2002–2003) and the Middle East (2012), respectively [4, 5]. On the other hand, four coronaviruses, namely, HCoV-229E and HCoV-NL63 of the alpha*-genus* and HCoV-OC43 and HCoV-HKU1 of the *beta genus*, have been revealed to cause mild respiratory complications in humans [2]. The International Committee on Taxonomy of Viruses identified its symptomatic and biological similarity with SARS-CoV, hence renamed the virus the severe acute respiratory syndrome coronavirus-2 (SARS-CoV-2), and the disease caused by this virus was renamed COVID-19 (coronavirus disease-19) by the World Health Organization (WHO). It is a positive stranded-RNA virus belonging to the sarbecovirus subgenus of the β-coronavirus, which also comprises other zoonotic viruses, such as SARS-CoV and bat SARS-like CoV [3]. Similar to other viruses, SARS-CoV-2 thrives on several intermediate and final hosts before infecting humans, which makes the prevention and treatment of viral infection more difficult. Although the virus has a lower mortality rate than SARS-CoV and MERS-CoV, it possesses high infectivity and transmissibility. A genome sequencing study of novel coronavirus showed that SARS-CoV-2 has 79.5% and 96% resemblance to SARS-CoV and SARSr-CoV-RaTG13 (bat SARS coronavirus), respectively, which implies the origin of coronavirus from a bat [6]. There have been several reports published on the discovery of a large number of SARS-related coronaviruses from bats, a natural reservoir of coronaviruses [7–9]. As of 20 July 2020, a total of 14,845,850 people have been infected, and 612,815 have died of the disease globally [10]. In Bangladesh, a total of 207,453 people have been infected by the virus and 2668 have died of the disease as of 20 July 2020, with a mortality rate of 1.29%. This article aims to discuss the epidemiological study, genomic features, diagnosis, prevention, and treatment scenario of Bangladesh.

## Main text

### Composition of the genome and molecular basis of pathogenesis

**Fig. 1:**
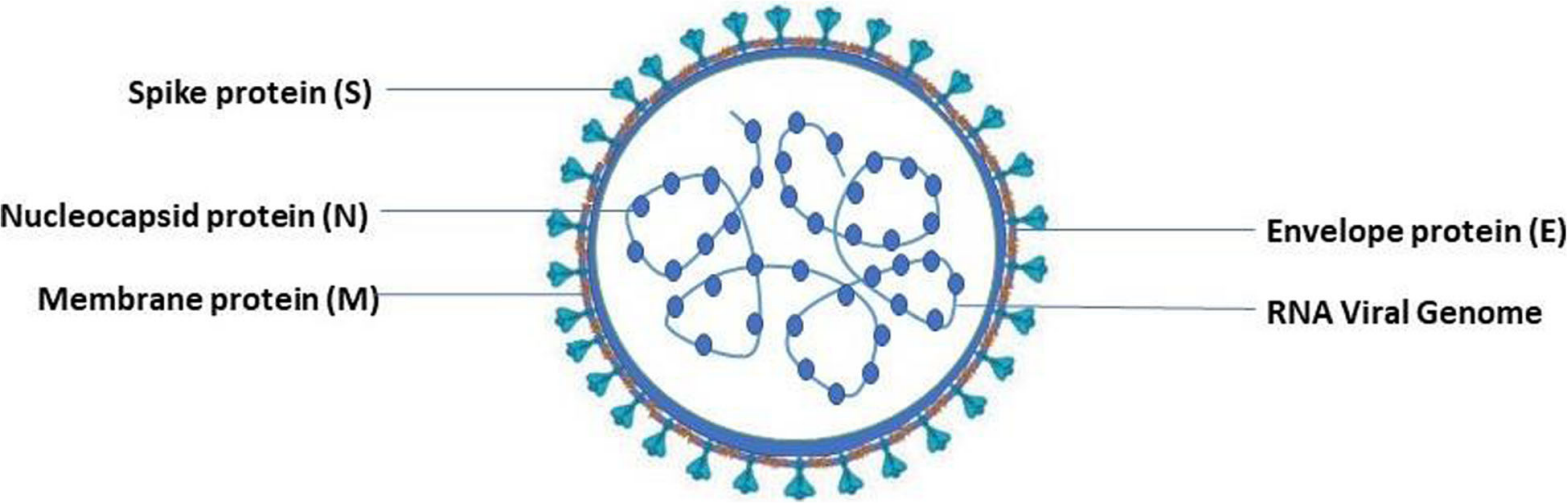
Structure of SARS-CoV-2

The genome of SARS-CoV-2 varies from approximately 26 to 32 kilobases and contains 14 open reading frames (ORFs). The longest ORF at the 5′ terminus that represents the major portion of the genome (67%) encodes 16 non-structural proteins, while the 3′ terminus encodes structural proteins [11]. Of them, the membrane protein (M), nucleocapsid protein (N), envelope protein (E), and spike glycoprotein (S) are the four main structural proteins shown in Fig. 1.

Moreover, its genome comprises eight accessory proteins, namely, 3a, 3b, p6, 7a, 7b, 8b, 9b, and orf1 [12]. There is an ample amount of M glycoproteins in the SARS-CoV-2 genome that are responsible for the formation of virus particles and conduce virus assembly. It consists of a small −NH_2_ terminal domain uncovered to the outside of the virion, three transmembrane domains, and a much longer −COOH terminus that resides inside the virion [13]. The N protein is the only protein present in the nucleocapsid. Although it is predominantly intricated in various viral genome-related processes, particularly in virus assembly and the formation of nucleocapsids, it is also involved in replication-transcription complexes in infected cells and hosts cellular responses to viral infection [14, 15]. The smallest structural protein E is involved in various processes related to virus replication cycles, such as virus assembly, budding, envelope formation, and pathogenesis [16]. The S protein mainly paves the way for the virus to bind and enter into the host cell membrane and results in infection. It consists of two subunits: the S1 subunit, which contains a signal peptide and the receptor-binding domain, and the S2 subunit, which contains conserved fusion proteins, a transmembrane domain and the cytoplasmic domain [17]. Hoffmann et al. demonstrated that the S protein of the virus, similar to SARS-CoV, has a strong propensity to bind with human angiotensin-converting enzyme 2 (ACE2) and targets ACE2 as a receptor for entry and undergoes structural changes to merge with the host [18]. However, SARS-CoV-2 is more dangerous because its affinity towards ACE2 is more than 10-fold higher than that of SARS-CoV [19]. Furthermore, novel coronavirus also utilizes the cellular protease TMPRSS2 (transmembrane protease, serine 2) because the S protein needs to be primed first before entering host cells. Generally, SARS-CoV-2 has less sequence homology with SARS-CoV (approximately 79%) and MERS-CoV (approximately 50%) [20].

Analysis of the genome sequence conducted by Lu et al. showed that its genome possesses 88% sequence homology with two bat coronaviruses, bat-SL-CoVZC45 and bat-SL-CoVZXC21 [20]. All the genome sequencing data of SARS-CoV-2 from all over the world are being uploaded to the Global Initiative on Sharing All Influenza Data (GISAID), which facilitates the process of analyzing and understanding its genomic biology. A total of 71,000 genomic sequences were available on the website until 24 July 2020 [21]. Following the entry and denudation of the virus, its genome commences the transcription process followed by the translation process. The replication and transcription processes of coronaviruses occur in the cytoplasm. The mechanism of replication requires continuous RNA synthesis to determine whether transcription involves discontinuous synthesis [22]. These mechanisms are stimulated by the replication-transcription complex encoded by a 20-kb replicase gene and presumed to consist of as many as 16 viral proteins and different proteins involved with cellular processes [23]. The expression of the replicase gene is stimulated by the translation of the genomic RNA. After an individual is exposed to a potential virus source such as bat or pangolins or persons infected by the virus, human antigen processing cells (APCs) and virus-specific T lymphocytes counter the entry of the virus. APC, along with human major histocompatibility complex (MHC) molecules known as human leukocyte antigens (HLAs), responds to and mediates defense against novel coronavirus attacks [24]. Genetic polymorphism in HLA genes elucidates why individual susceptibility to novel coronavirus varies in a diverse population, and this difference among individual susceptibility is also promoted by genetic polymorphisms in mannose-binding lectin (MBL).

### The genome variants of SARS-COV-2 strain from Bangladesh

The first complete genome sequence of SARS-CoV-2 (CHRF_nCoV19_0001) from a Bangladeshi isolate was submitted to the GISAID database on 12 May 2020. Bangladesh-based Child Health Research Foundation conducted the sequencing project [25]. As of 17 July, genome sequencing of 222 samples from Bangladeshi SARS-CoV-2 isolates was submitted on the GISAID database, of which 173 samples were sequenced by the Bangladesh Council of Scientific and Industrial Research (BCSIR) [26]. Jashore University of Science and Technology had announced on 24 June 2020 that they had sequenced the complete genome of another three SARS-CoV-2 strain that is responsible for the respiratory infections in the southern part of the country [27]. A comparative study of the Bangladeshi strain and other strains around the world revealed that there were 9 single-nucleotide variants in the Bangladeshi strain, mostly in the ORF1ab gene and also a sequence containing 3 successive variants in the N protein which probably reflects a weaker strain of SARS-CoV-2 and explain the reason of low mortality rate in Bangladesh [28]. Phylogenetic analysis elucidated that there is sequence homology among the Bangladeshi strains and strains from Taiwan, Greece, and Kazakhstan which implicates that the virus strains found in these countries were descendent of a weaker strain of the same origin, probably Michigan or Arizona in the USA [28]. Another investigation of 184 genome samples of SARS-CoV-2 across the country found 634 mutations located in the whole genome that results in 274 nonsynonymous substitutions in 22 different proteins. Among the spike protein variants circulating across the country, G614 is the most prevalent followed by L323 (94%) in RNA-dependent RNA polymerase (RdRp), K203 (82%) and R204 (82%) in nucleocapsid, and F120 (78%) in NSP2. These mutations implicated a close homology with a European haplotype of SARS-CoV-2. These sequencing data will help to predict prognosis and develop an effective vaccine for the treatment of COVID-19 patients in Bangladesh.

### Epidemiology of COVID-19 in Bangladesh

**Fig. 2:**
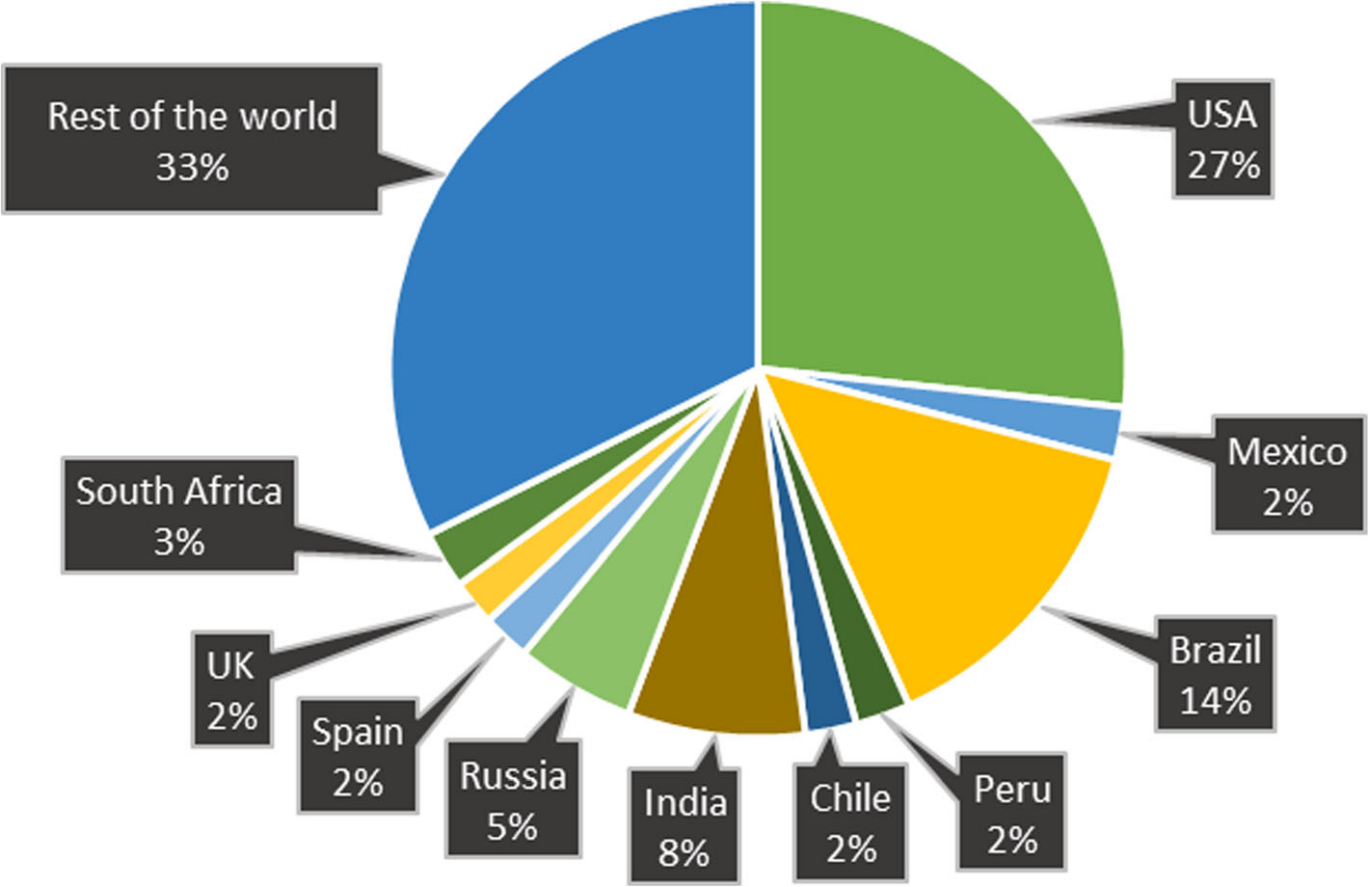
Global distribution of confirmed cases of the COVID-19 pandemic

The novel coronavirus outbreak was announced as a public health emergency of international concern on the 30th of January 2020 by the World Health Organization (WHO) and as a controllable pandemic on the 11th of March [17]. As of 20 July 2020, a total of 14,845,850 people had been infected by the virus and 612,815 people had died of the disease globally [10]. The worst-hit country being the USA with almost 4 million confirmed cases which constitutes almost 27% of the total cases and 143,834 fatalities. In Europe, Russia was the country with the highest number of confirmed cases by 20 July 2020; a total of 777,486 people were infected and 12,427 persons had died. The global distribution of confirmed COVID-19 cases has been illustrated in Fig. 2 [10].

According to epidemiologic data, Yemen had the highest mortality rate around the world (27.7%) followed by France (17.3%) and UK (15.4%) until 20 July 2020 [29]. Since there is no identified preexisting immunity against the virus, the infection rate is increasing in an unpredictable manner and all individuals are vulnerable to the virus. One of the alarming news is that since SARS-CoV-2 is an RNA virus, it continuously mutates and even after taking preemptive precautions and measures, the number of infected cases is increasing.

Bangladesh has become the new hotspot of COVID-19 and has the highest number of infected patients among the South Asian countries after India and Pakistan. According to the Institute of Epidemiology, Disease Control and Research (IEDCR), so far, 207,453 people have been infected and 2668 have died of the disease in Bangladesh until 20 July 2020, with a mortality rate of 1.29%. Around the same time, 113,558 COVID-19 patients have recovered from the disease with a recovery rate of 54.8% [30]. The geographical distribution of confirmed cases was available on 70% of cases (144,281/207,453). Dhaka division has the highest number of infected patients in the country with more than 50% of all reported cases, of which 48,322 have been reported from the country’s capital Dhaka city. Chattogram (29,661) and Rajshahi (10,477) divisions positioned the second and third places regarding most confirmed COVID-19 patients, respectively [30]. Of the 2668 deceased cases, the highest number was also reported from the Dhaka division (1305), followed by the Chattogram division (673) and the Khulna division (173) (Table 1) [30, 31]. The country reported its first confirmed SARS-CoV-2 infection on 8 March and its first death on 18 March 2020. In a 24-h period, the highest 4019 cases were reported on 2 July and the highest 64 fatalities on 30 June [32]. It saw a rapid rise of infection at the middle of May; almost 90,038 confirmed cases and 1136 deaths have been reported between week 21 and week 25 which is more than the total number combined in the initial days, albeit infection had been on a steady increase in the initial month [30]. The country reached 10,000 cases on the 3rd of May, exceeded 100,000 confirmed cases on 18 June, and has recently crossed 200,000 infected patients on 18 July.

**Table 1:** COVID-19 confirmed cases in Bangladesh (up to 20 July 2020)

Division	Total confirmed case	Percentage	Total deaths	Percentage
Dhaka	77,354	53.61	1,305	48.91
Chattogram	29,661	20.56	673	25.22
Rajshahi	10,477	7.27	144	5.40
Khulna	9483	6.58	173	6.49
Sylhet	6672	4.62	125	4.69
Barisal	4021	2.79	100	3.75
Mymensingh	3687	2.55	58	2.17
Rangpur	2926	2.02	90	3.37

**Fig. 3:**
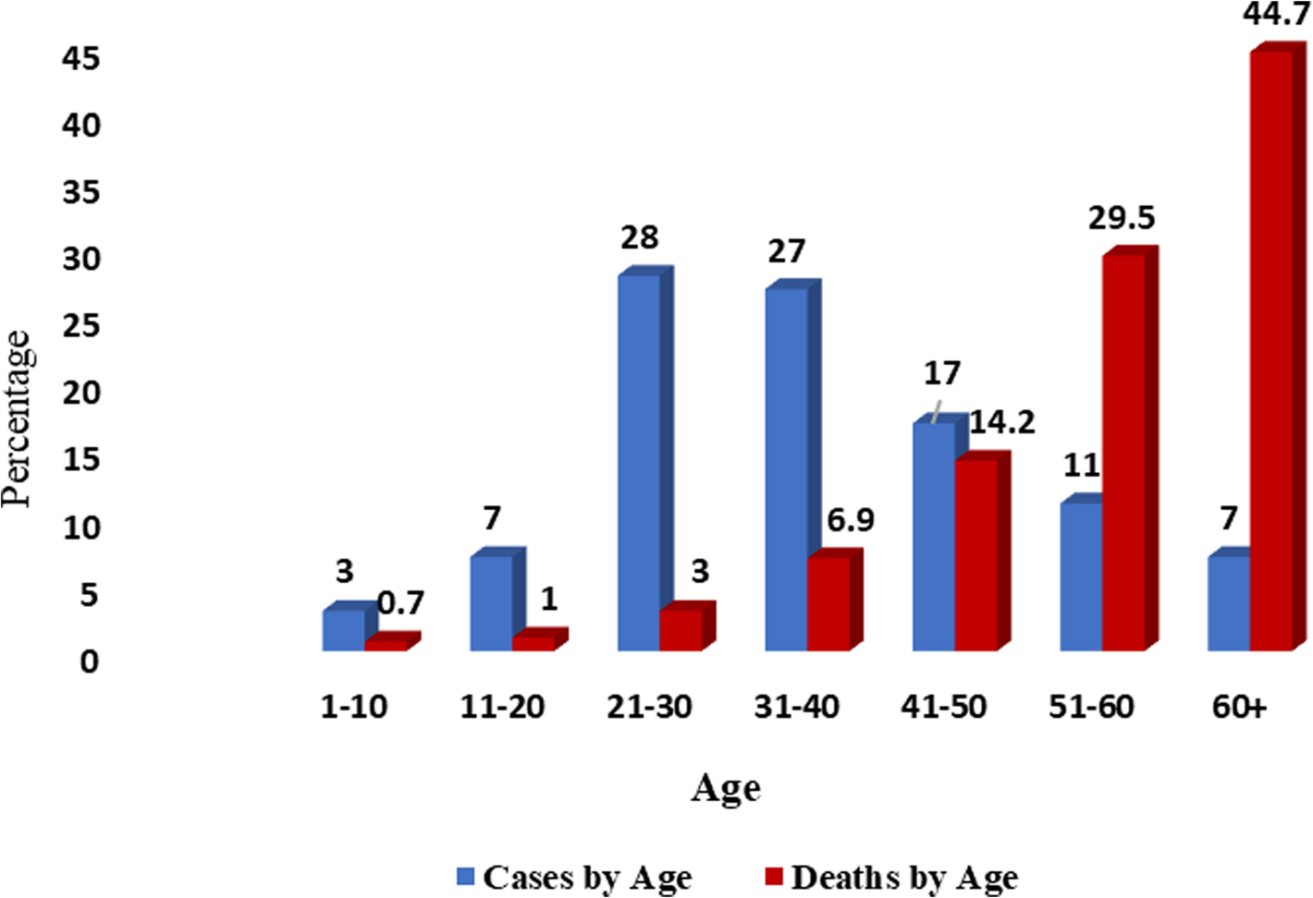
Case and death percentage by ages in Bangladesh

On 20 July 2020, the COVID-19 attack rate, i.e., AR (the total number of cases/the total population), in Bangladesh was 1218.1 per 1 million and all of the 64 districts have been infected by SARS-CoV-2. The highest AR was observed in the Dhaka division (3182.6/1,000,000), the most populous division of the country, and within the division, the country capital Dhaka has the highest AR (almost 12,915.9/1,000,000) [33]. A study showed that the mean reproduction number of COVID-19 in Bangladesh until 25 June 2020 is 1.40 (95% Cl 1.11, 1.73) [34]. Analysis of demographic data of COVID-19 patients elucidated that, though more than 50% of patients infected by SARS-CoV-2 in Bangladesh are aged between 21 and 40, the highest mortality rate was observed in elderly people aged more than 60 years as they have a relatively weaker immune system (Fig. 3) [30, 35].

**Fig. 4:**
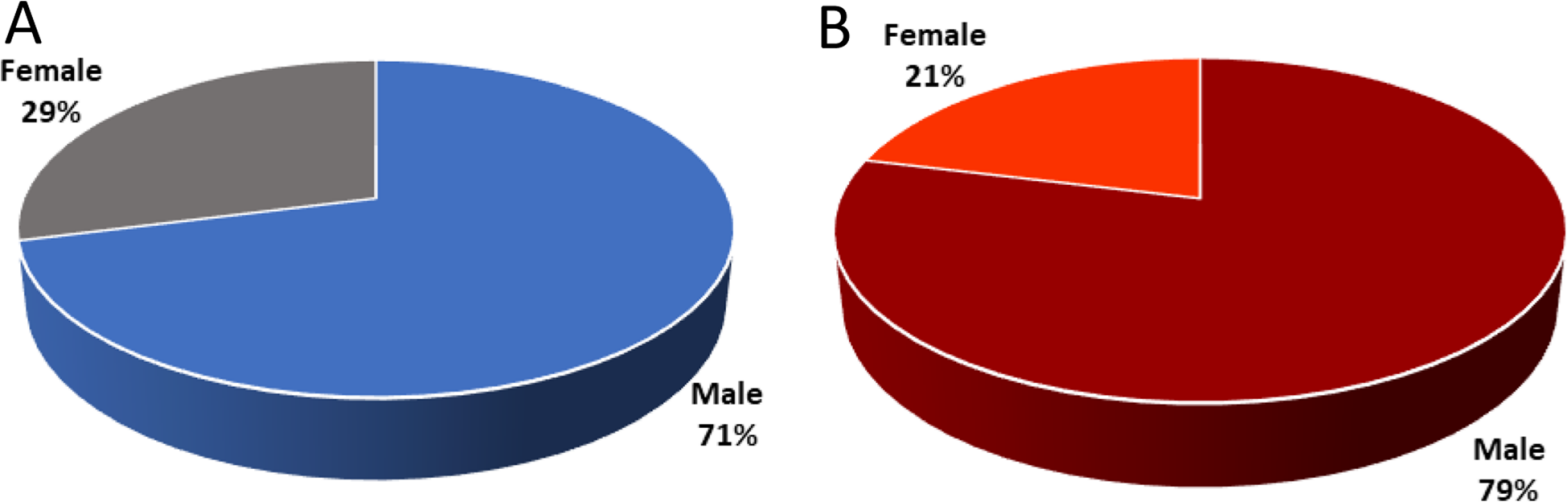
Percentage by gender in Bangladesh. **a** Cases. **b** Deaths

Male individuals were more susceptible to the virus than females and consist of 71% and 29% confirmed cases, respectively (Fig. 4a). Among the deceased, 2104 were male and 564 were female [35]; the death ratio of males to females was 79:21 (Fig. 4b).

Out of the 116,226 cases with known outcomes in Bangladesh by 20 July 2020, the mortality rate is almost 2.3% (2668/116,226) which is comparatively lower than the global averages of 6.4% (612,815/9,514,219) [10, 30]. SARS-CoV-2 infection to the health professionals has been another problem for Bangladesh because there are only 93,358 MBBS doctors and 9569 BDS doctors in the country according to the recent data of DGHS [36]. As of 18 July 2020, there were a total of 5690 infected cases among healthcare professionals including 1995 doctors, 1536 nurses, and 2159 other personnel and, of them, 67 doctors died from the disease [37]. Law enforcement personnel are also at risk of SARS-CoV-2 infection as they are engaged in maintaining government injunction, and thus, 13,316 policemen have been infected by the virus, and 53 of them have died till the 17th of July 2020. Yet 10,076 cops have already cured of the disease and returned to their respective station [38]. Rohingya refugees living in Cox’s Bazar district are also vulnerable populations to infectious disease because more than 860,000 Rohingya refugees dwell in such a congested place [39]. As of 19 July 2020, a total of 62 COVID-19 confirmed cases and 6 related deaths have been reported in Rohingya camps. But the exact patients may be more because so far only 1288 tests have been conducted among the population [40].

### Clinical features of COVID-19 among Bangladeshi patients

Though the incubation period of SARS-CoV-2 was reported to be 2–10 days according to WHO [41], it demonstrated to infect people with a median incubation period of 3 days [25]. The maximum latency was observed up to 24 days which is longer compared to MERS (5 days) and seasonal flu (2 days). This implies the necessity for longer periods of quarantine or effective monitoring of persons potentially exposed to the pathogen [26]. The disease caused by the SARS-CoV-2 manifests with diverse clinical characteristics ranging from asymptomatic patients to acute pneumonia with multiorgan failure. After analyzing various symptoms, the disease is now classified into four levels of severity [27]. Mild patients only exhibit symptoms of viral infection in the upper respiratory tract while moderate patients manifested with fever, respiratory symptoms of cough, and shortness of breath [28]. Patients with severe cases manifest with severe dyspnea, acute respiratory distress, tachypnea (≥ 30 breaths/min), and blood oxygen saturation (SpO_2_ ≤ 93%). Finally, patients who need ICU support are considered as critical and show features such as respiratory arrest, septic shock, and multiple organ dysfunction or failure [42, 43].

A study of 103 RT-PCR-confirmed COVID-19 Bangladeshi patients demonstrated that 74.76% were mild, 9.71% were moderate, and 15.53% were severely ill patients. The most predominant clinical features include fever (78.6%), fatigue (68%), cough (44.7%), loss of appetite (37.9%), shortness of breath (37.9%), and anosmia (35.0%) [44]. Another study of 100 COVID-19 patients demonstrated symptoms like rhinitis (13.0%), body ache (13%), headache (12.0%), sputum (7.0%), abdominal pain (4.0%), and hemoptysis (3.0%) [45]. A cohort study of 201 patients showed that very few patients were manifested with rare symptoms like burning body (1%), toothache (1%), itchiness (0.5%), red-eye (0.5%), oral ulcer (0.5%), and constipation (0.5%) and 4.5% were asymptomatic [46]. Hypertension (34.0%) was the most prevalent comorbidities associated with the patients followed by diabetes mellitus (21.4%). Ischemic heart disease (9.7%), chronic kidney disease (7.8%), renal disease (8.0%), and asthma/COPD (6.0%) were also manifested in the patients [44]. Anosmia is another prominent feature of COVID-19 and can appear suddenly as the sole symptom of the COVID-19 patients immediately after the onset of disease [47, 48]. This symptom was present in 39% of mild, 40% of moderate, and 12.5% of severe Bangladeshi patients [44]. Findings from a study of 30 pregnant women with confirmed COVID-19 infection showed that abdominal pain (63.3%) was the predominant symptom followed by dry cough and fever. Nearly 16.7% of the patients were asymptomatic and two women out of the 30 women had died (6.7%) indicating a relatively lower mortality rate among pregnant women [49]. Clinical manifestations of the patients of COVID-19 have many similarities with the patients of SARS and MERS (Table 2).

**Table 2:** Clinical features of SARS, MERS, and COVID-19

SARS	MERS	COVID-19
1. Predominant features were constant fever, chill or rigors, dry cough, myalgia, headache, and malaise [50].	1.Onset of disease with fever, chills or rigors, cough, sore throat, myalgia, and arthralgia, followed by dyspnea [51, 52].	1. Predominant symptoms are fever, cough, and sore throat. Other common features are fatigue, myalgia, and dyspnea [53, 54].
2. Less common features were sore throat, running nose, sputum production, dyspnea, anorexia, nausea and vomiting, headache, and dizziness [50, 55].	2. Enteric manifestations such as diarrhea, vomiting, anorexia, and stomach pain [56, 57].	2. Unusual features such as headache, nausea or vomiting hemoptysis, and diarrhea [1, 25].
3. Severe cases: recurrence of fever, shortness of breath, hypoxemia, and ARDS [58].	3.Severe patients: ARDS, multiple organ failure including renal failure and respiratory failure [59, 60].	3. Severe patients: acute pneumonia, hypoxemia, and ARDS [28].
4. Almost 61% manifested mild symptoms, 11.1% had acute respiratory complications [27].	4. 21% of patients exhibited mild or no symptoms, whereas 46% possess severe complications or died [61].	4. 80.9% of patients had mild pneumonia, 13.8% had acute complications, and 4.7% considered critical and needed ICU care [62].

Laboratory findings of 168 critically ill Bangladeshi patients showed that nearly 70% of the patients exhibited elevated white blood cells, neutrophils, and D-dimer [63]. Another retrospective study found that increased levels of glucose, ferritin, C-reactive protein, and erythrocyte sedimentation rate (ESR) were also common among the patients. An elevated level of troponin was also observed among patients and it is hypothesized to be due to myocarditis, microangiopathy, myocardial infarction, and other cardiovascular diseases [64–67].

### Prevention and diagnosis scheme of COVID-19 in Bangladesh

The government of Bangladesh announced the first lockdown on 26 March and extended it to 16 May [68]. On 16 March, the government announced to shut down all the educational institutions from 17 to 31 March and later declared to continue the closure up to September until the infection rate has considerably decreased [69]. After that, the government lifted the lockdown for a few days and saw a massive increase in both infected and death numbers. The government has planned to impose a zone-wise lockdown from the 9th of June under a pilot project. According to the plan, infected areas will be divided into 3 zones depending on COVID-19 severity: red zones—strict lockdown will be maintained to decrease the spreading of the disease and no person is allowed to cross the zone, yellow zones—limited restriction will be imposed on the regular life, and green zones—where virtually no or very few COVID-19 patients exist [70]. The country suspended all international travel on 5 April, except for flights to and from China [71]. The government could not impose strict social distancing measures, especially in Dhaka where lives almost 1.1 million slum dwellers, earning their day-to-day livelihood [72]. Moreover, the majority of these slum dwellers have no knowledge of the threat posed by COVID-19, and they live in an unhygienic condition with very little supply of water and one bathroom for every 10–16 families that made them more vulnerable to infectious disease [73].

**Fig. 5:**
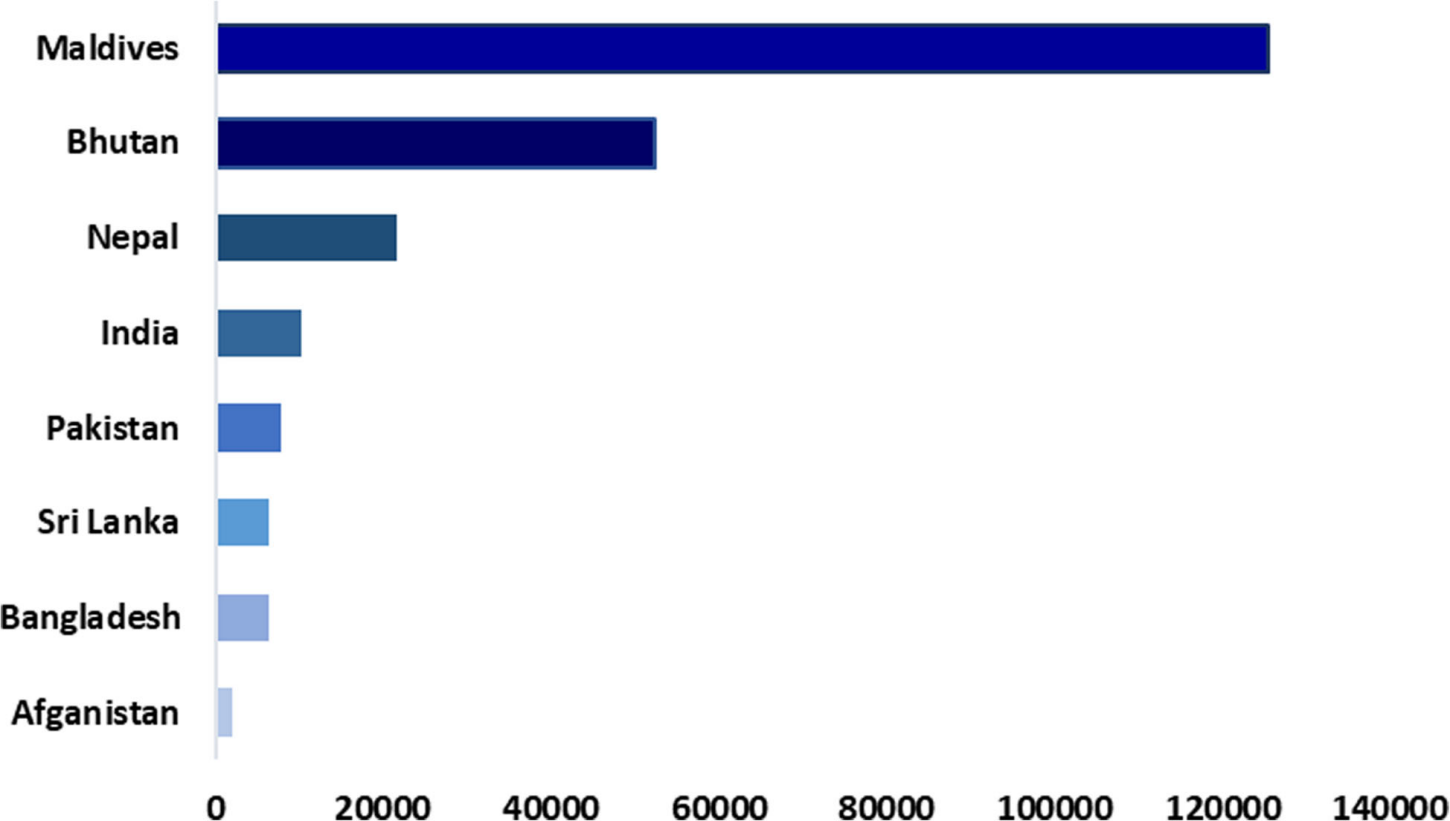
Test per 1 million people in the South Asian countries

Currently, 80 public and private laboratories (46 in Dhaka and 34 outside of Dhaka) including several public universities that have real-time PCR machines have been conducting COVID-19 tests [74, 75]. A total of 1,041,661 tests was conducted by 20 July 2020, with an overall positivity rate of 20% and almost 61.3% (638,258/1,041,661) tests were done in the laboratories of Dhaka city [32]. Although the number of testing has been elevating day by day, it is still too far from reaching the target number required to test every possible suspected case, and among the South-Asian countries, Bangladesh was placed only above Afghanistan regarding COVID-19 testing. Only 6322 per 1 million testings have been done until 20 July 2020 (Fig. 5) [10, 76].

Another diagnostic testing kit was developed by Gonoshasthaya Kendra, Bangladesh, on 17 March 2020. This cost-effective kit (only $3.25 to produce) named “Gonoshasthaya Rapid Dot Blot” can test antigen as well as antibody using saliva and swab samples and yields results within 15 min. The team led by Dr. Bijon Kumar Sil had been waiting for approval from the Directorate General of Drug Administration (DGDA) to use their kit immediately [77, 78]. In recent clinical trials, the Gonoshasthay’s kit has shown 97.7% sensitivity and 96% specificity in internal validation that satisfies the US FDA guidelines for antibody testing kits [79].

Radiographic images have been analyzed to diagnose and facilitate early detection of this novel coronavirus disease. Chest computed tomography (CT) is being used as an important complementary tool to the RT-PCR tests to detect the viral infection. Analysis of several RT-PCR assays and CT scans illustrated that the sensitivity of chest CT for SARS-CoV-2 was 97% [80]. Nearly 60–93% of patients displayed initial positive chest CT results with pneumonia before the initial positive RT-PCR results and follow-up CT scans showed that 42% of patients demonstrated improvement prior to the RT-PCR results turn out to be negative [81]. Chest CT findings of 114 Bangladeshi SARS-CoV-2-infected patients illustrated ground glass opacities without consolidation for 91.2% (104) patients and with consolidation for 44% patients. Bilateral chest CT (94%), vascular thickening (66.7%), crazy paving appearance, and fibrotic shadows were also common [82]. Another study of 128 RT-PCR positive patients found positive HRCT results in 123 patients, where most of the patients (75%) had numerous lesions involving all 5 lobes of the lung. The lesions were predominantly present in the periphery region (96%) and posterior region (80.5%) [83]. Manifestation and degree of lung involvement observed by chest CT can assist to ascertain treatment strategies for the severe and critical COVID-19 patients.

### Treatment scenario of COVID-19 in Bangladesh

So far, 91,229 patients are currently under treatment in different medical institutes, and 43,007 patients were placed in isolation units as of 20 July 2020. Currently, the country has 629 institutional quarantine centers that can accommodate 31,991 patients across 64 districts, and as of 20 July, 23,331 individuals were gone under institutional quarantine and of them, 17,406 have got release while 335,643 out of 389,150 individuals have been released from home quarantine [76]. Different antiviral drugs and treatment strategies have been implemented to treat COVID-19 patients. DGHS recommended controversial drugs such as chloroquine and hydroxychloroquine for the treatment in the “National Guidelines on Clinical Management of Coronavirus Disease-2019” [84]. A medical team from Bangladesh Medical College Hospital claimed that a combination of antiprotozoal medicine ivermectin and antibiotic doxycycline was effective for the treatment of 60 COVID-19 patients [85]. Clinical trials are going on and until now 400–500 COVID-19 patients have received the drugs, of them almost 98% of patients had recovered within 4–14 days [86]. Bangladesh-based Beximco Pharmaceuticals had launched the world’s first generic remdesivir—an antiviral drug for the treatment of COVID-19 patients on 21 May 2020 [87]. In a recent clinical trial, it has demonstrated potential effectivity against SARS-CoV-2 and lessened the recovery time [88]. Remdesivir which works by inhibiting the replication of SARS-CoV-2 has now become the talk of the town and recently received “Emergency Use Authorisation” from the FDA and developed by Gilead Sciences [89]. Bangladeshi-based pharmaceutical Globe Biotech Limited has announced on 1 July 2020 that they are developing a COVID-19 vaccine which showed effectivity on preliminary trials on animal models [90]. They have also developed a guideline for clinical trials which will be conducted after getting approval from the Bangladesh Medical Research Council (BMRC) and claimed to produce the vaccine within 6 months after clinical trials. The research group of 12 scientists analyzed 5743 genome sequences of the SARS-CoV-2 from the US National Center for Biotechnology Information (NCBI) through the bioinformatics approach and conducted the test through the surface plasmon resonance (SPR) method to detect molecular interactions [91, 92]. The BMRC has recently approved the phase III clinical trials of a candidate vaccine developed by Sinovac Research and Development Co Ltd, China in Bangladesh. The trials will involve 4200 volunteers at seven different hospitals and will begin in the early of August this year under the supervision of the International Centre for Diarrhoeal Disease Research, Bangladesh (icddr,b) [93, 94]. The country has recently started clinical trials of convalescent plasma therapy (CPT) and received ethical approval from DGDA and the BMRC. Eighteen patients were administered plasma from recovered patients, and the government in collaboration with several national organizations has recently inaugurated a plasma network namely “Shohojoddha” to consolidate plasma therapy across the country [95, 96].

### Economic impacts of COVID-19 on Bangladesh

Bangladesh likewise most of the countries of the world is facing the gravest economic crisis for this unprecedented pandemic. Export Promotion Bureau of Bangladesh (EPB) revealed that export earnings have fallen to 520 million USD in March this year from 3.03 billion USD in the same month of the previous year [97]. The International Monetary Fund (IMF) had projected the country’s GDP growth to be 2–3.8% for the fiscal year 2019–2020 from 7.4–8.2% which was estimated before [98]. According to the Bangladesh Bureau of Statistics (BBS), currently, thirty-four million people live below the poverty line that constitutes 20.5% of the total population. A survey conducted by the South Asian Network on Economic Modelling (SANEM) denoted that if the income level for the poverty line is increased 1.25% the number of poor people would be raised to thirty-six million [99]. The prime minister of Bangladesh had declared to provide 19 incentive packages of Tk 1.03 trillion (equivalent to 12.13 billion USD) to cushion the economic blow of the COVID-19 pandemic [100]. She also announced an incentive package of 600 million dollars to facilitate the industries dealing with exporting products [101]. But the government alone cannot confront this unprecedented situation and requires both public and private collaboration. Co-operation between the local as well as international organizations such as the World Health Organization and the World Economic Forum is mandatory to combat this massive economic blow caused by COVID-19.

### Points to be considered in Bangladesh

- Zone-wise lockdown should be continued, and the government should keep in mind both the economic challenges and COVID-19 infection rate.
- Implementation of a nationwide contact tracing is mandatory, which will help to locate the infected individuals and their contacted persons and prevent secondary infections.
- Both the national and international public health agencies should come together for scaling up surveillance operations throughout the country.
- Uses of low-cost and rapid diagnostic kits (such as Gonoshasthay’s kit) would be an effective strategy to identify all possible suspected cases which will mitigate the spreading.
- Finally, raising campaigns about using face masks, maintaining social isolation, and eliminating the rumors of COVID-19 among the mass population can be some possible measures.

## Conclusion

The ongoing COVID-19 pandemic caused by this newly emerged virus is undoubtedly a matter of international health concern. Investigation of viral genomic, pathogenesis and epidemiological study have been continued to find potential treatment strategies. In Bangladesh, delay of strategic planning, scarcity of healthcare facilities (PPE, ventilators), shortage of appropriate preventive measures (quarantine and isolation unit), emergency service, unawareness, and lack of knowledge of the people about the COVID-19 is aggravating the situation. As a developing country, Bangladesh should focus on scaling up its readiness and response operation as well as improving the healthcare facilities such as upgrading the capacity of the testing laboratories, institutional quarantine centers, and stocking personal protective equipment. Promoting knowledge and awareness of the COVID-19 among the mass people is also significant.

## Data Availability

All data and material are available upon request.

## Abbreviations

COVID-19: Coronavirus disease 2019
SARS-CoV-2: Severe acute respiratory syndrome coronavirus 2
HCoV-229E: Human coronavirus 229E
HCoV-OC43: Human coronavirus OC43
HCoV-NL63: Human coronavirus NL63
HCoV-HKU1: Human coronavirus HKU1
SARS-CoV: Severe acute respiratory syndrome coronavirus
MERS-CoV: Middle East respiratory syndrome coronavirus
WHO: World Health Organization
SARS: Severe acute respiratory syndrome
MERS: Middle East respiratory syndrome
ACE2: Angiotensin-converting enzyme 2
TMPRSS2: Transmembrane protease, serine 2
GSAID: Global Initiative on Sharing All Influenza Data
MHC: Major histocompatibility complex
SNP: Single-nucleotide polymorphism
ARDS: Acute respiratory distress syndrome
CT: Computed tomography
RT-PCR: Reverse-transcription polymerase chain reaction
CDC: Centers for Disease Control and Prevention
IEDCR: Institute of Epidemiology, Disease Control and Research
DGHS: Directorate General of Health Services
AR: Attack rate
DGDA: Directorate General of Drug Administration
BMRC: Bangladesh Medical Research Council
EBP: Extracorporeal blood purification
