# Correction to: A review of the genome, epidemiology, clinical features, prevention, and treatment scenario of COVID-19: Bangladesh aspects

**DOI:** 10.1186/s43168-021-00057-y

**Published:** 2021-02-08

**Authors:** Abdullah Al Noman, Md. Shofiqul Islam, Samiron Sana, Prapti Mondal, Rima Islam Meem, Sohel Rana, Debashish Mondol, Manoshi Sana, Sheikh I. Hossain, Taufique Joarder, Kishor Mazumder

**Affiliations:** 1 Department of Genetic Engineering & Biotechnology, Faculty of Biological Science and Technology, Jashore University of Science and Technology, Jashore, 7408 Bangladesh; 2 Pharmacy Discipline, Life Science School, Khulna University, Khulna, 9208 Bangladesh; 3 Department of Pharmacy, Faculty of Biological Science and Technology, Jashore University of Science and Technology, Jashore, 7408 Bangladesh; 4 Department of Zoology, Rajshahi College, Rajshahi, 6100 Bangladesh; 5 Dhaka Medical College, Dhaka, 1000 Bangladesh; 6 School of Mechanical and Mechatronic Engineering, University of Technology Sydney, 81 Broadway, Ultimo, NSW 2007 Australia; 7 Public Health Foundation, Dhaka, Bangladesh; 8 School of Biomedical Sciences, Charles Sturt University, Wagga, NSW 2678 Australia

**Correction to: Egypt J Bronchol (2021) 15:8**

**https://doi.org/10.1186/s43168-021-00053-2**

After publication of the original article [1], the authors identified an error in the author’s name of Sohel Rana.

The incorrect author name is: Shohel Rana.

The correct author name is: Sohel Rana.

The original article has been corrected.
